# Chemo-Immunotherapy Using Lentinan for the Treatment of Gastric Cancer with Liver Metastases

**DOI:** 10.3390/medsci4020008

**Published:** 2016-04-07

**Authors:** Kenji Ina, Ryuichi Furuta, Takae Kataoka, Satoshi Kayukawa, Hiroko Ina, Masahiko Yoneda

**Affiliations:** 1 Department of Medical Oncology, Nagoya Memorial Hospital, 4-305 Hirabari, Tenpaku-ku, Nagoya 468-8520, Japan; syoukaki@trust.ocn.ne.jp; 2 Department of Clinical Oncology, Nagoya Memorial Hospital, Nagoya 468-8520, Japan; t-kataoka@hospy.or.jp (T.K.); kayu-kawa@i.softbank.jp (S.K.); 3 School of Nursing and Health, Aichi Prefectural University, Nagoya 463-8502, Japan; betty_chan1019@yahoo.co.jp (H.I.); yoneda@nrs.aichi-pu.ac.jp (M.Y.)

**Keywords:** lentinan, trastuzumab, gastric cancer, liver metastases, chemo-immunotherapy

## Abstract

Gastric cancer is the third leading cause of cancer-related mortality worldwide. Systemic chemotherapy is the main treatment option for advanced gastric cancer when the tumor is inoperable. Despite recent advances in chemotherapeutic agents, the prognosis of unresectable or recurrent gastric cancer remains extremely poor. In Japan, combination therapy including S-1 and cisplatin is the standard first-line treatment for advanced gastric cancer; however, the five-year survival rate remains very low. Lentinan, the backbone of beta-(1,3)-glucan with beta-(1,6) branches, an active ingredient purified from *Shiitake* mushrooms, has been approved as a biological response modifier for the treatment of gastric cancer. This agent has been used in combination with oral fluoropyrimidines to improve the overall survival of gastric cancer patients. A retrospective chart review on 138 metastatic gastric cancer patients receiving chemotherapy was performed in Nagoya Memorial Hospital from 1 September 2010 to 31 August 2015. 12 patients with liver metastases were treated by lentinan in combination with S-1-based chemotherapy. The rate of objective response was 42% (5/12) and the disease control rate was 83% (10/12) in response to chemo-immunotherapy using lentinan, with a median overall survival of 407 days (95% CI: 207–700 days).

## 1. Introduction

Gastric cancer is one of the most common neoplasms and the third leading cause of cancer-related mortality worldwide [1]. Systemic chemotherapy is the main treatment option for advanced gastric cancer when the tumor is inoperable [2]. Despite recent advances in chemotherapeutic agents, the prognosis of advanced gastric cancer remains poor with a median overall survival (OS) of one year [2,3]. The presence of liver metastases especially showed the worst survival among the gastric cancer patients receiving chemotherapy [4]. In Japan, a combination of S-1, an oral derivative of 5-fluorouracil, and cisplatin is considered the standard first-line treatment for unresectable or recurrent gastric cancer [3]; however, the five-year survival rate remains very low [5]. Recent clinical studies have shown that chemo-immunotherapy using lentinan prolongs the survival of gastric cancer patients, compared to cytotoxic chemotherapy [6,7]. Lentinan, an active ingredient purified from *Shiitake* mushrooms, has been approved for use as a biological response modifier in the treatment of gastric cancer [8,9]. Beta-glucans stimulate macrophage to produce cytokines such as IL-12 and in turn activate adaptive immunity. The administration of lentinan was reported to enhance the antigen-presenting functions of dendritic cells, thereby inducing tumor-specific cytotoxic T cells [10]. Lentinan also upregulated the NK-cell-mediated killing of tumor cells [11]. Since T-cell function as well as NK activity are suspected to be downregulated in cancer patients, the usage of this beta-glucan might restore the host immune responses. Aiming at improving OS, lentinan was administered to gastric cancer patients with multiple liver metastases in combination with S-1-based chemotherapy.

## 2. Patients and Methods

A retrospective chart review on metastatic gastric cancer patients receiving chemotherapy was performed at Nagoya Memorial Hospital from 1 September 2010 to 31 August 2015. The original regimens of cytotoxic chemotherapy include S-1 monotherapy, S-1/cisplatin [3,5], and PSC triple therapy [12] (Table 1). 2 mg of lentinan was administered every 2 weeks in combination with chemotherapy. The objective response to chemotherapy was evaluated using the criteria proposed by the Japanese Research Society for Gastric Cancer for the primary lesion [13] and using the Response Evaluation Criteria in Solid Tumors (RECIST version 1.1) [14] for metastatic lesions. The disappearance of all evidence of cancer for at least 4 weeks was considered a complete response (CR). According to the RECIST, at least a 30% decrease in the sum of diameters of target lesions was considered a partial response (PR). The development of a new lesion or at least 20% increase in the sum of diameters of target lesions was defined as progressive disease (PD). Patients who did not satisfy the criteria for any of these categories were considered to have stable disease (SD). Disease control was defined as CR, PR, or SD. OS was calculated from the start of chemo-immunotherapy until death or the most recent follow-up day among the gastric cancer patients with liver metastases receiving lentinan. The Kaplan-Meier method was used to plot OS curves and then OS rates were compared by means of the log-rank test [3,5]between the patients showing objective response (CR and PR) and those without objective response (SD and PD). The National Cancer Institute common toxicity criteria version 4.0 was applied to evaluate adverse effects. Doses were adjusted at the initiation of subsequent cycles, if severe toxicity (grade 3–4) was present.

## 3. Results

Chemotherapeutic agents were administered in 138 patients for the treatment of metastatic gastric cancer. The characteristics of 12 patients with liver metastases who received lentinan in combination with chemotherapy are summarized in Table 1. There were eight men and four women, with a median age of 67 (range, 42–82) years. Performance status was 0 in 5 patients, 1 in 3, and 2 in 4. High expression of human epidermal growth factor receptor 2 (HER 2) was seen in two cases, and HER2-positive rate was 16.5%. Primary gastric lesions were resected in 3 patients at the time of diagnosis of liver metastases. Metastatic lesions other than those in the liver were identified in the lung in two patients, peritoneum in four patients, and lymph nodes in all patients. The overall response rate was 42% (5/12; CR in 1, PR in 4), and the disease control rate was 83% (Table 2). The median OS of 12 cases was 407 days (95% CI: 207–700 days). When comparing the patients showing objective response (*n* = 5) with those with SD or PD (*n* = 7), OS was significantly prolonged in the former group (*p* < 0.05). As for the chemotherapeutic regimen, six patients were treated with triple combination chemotherapy consisting of paclitaxel, S-1, and cisplatin (PSC regimen) [12] combined with lentinan. One CR was observed (#1), where oral fluoropyrimidines were stopped due to severe degree of skin adverse events. In the literature, CR has been noted in four cases, with a disappearance of the primary lesion and liver metastasis in response to chemotherapy [15,16,17,18] (Table 3). Before chemotherapy, a 72-year old man had multiple metastases to both hepatic lobes (H3), whereas the other three had liver metastasis limited to one lobe (H1). The patient with H3 achieved CR, but experienced recurrence 20 months after the start of chemotherapy [15]. In our CR case, multiple liver metastases (H3) as well as primary gastric lesion completely disappeared by chemo-immunotherapy using lentinan. This case has not experienced any recurrence for 33 months. In our series, only two patients (#4 and 5) showed high HER2 expression (Table 2). The molecular targeting agent, trastuzumab, was administered to both HER2-positive cases in combination with chemo-immunotherapy, which resulted in PR.

## 4. Case Report

**Figure 1:**
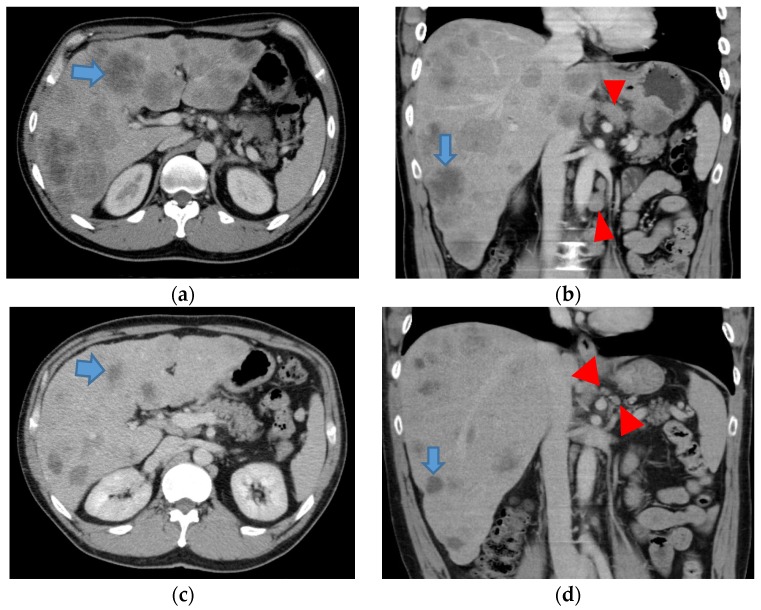
CT scan showed multiple liver tumors and swellings of lymph nodes before the start of chemo-immunotherapy with trastuzumab (**a**,**b**). Liver tumors (**blue arrows**) and lymph nodes (**red arrow heads**) remarkably decreased in size after four cycles of treatment (**c**,**d**).

**Figure 2:**
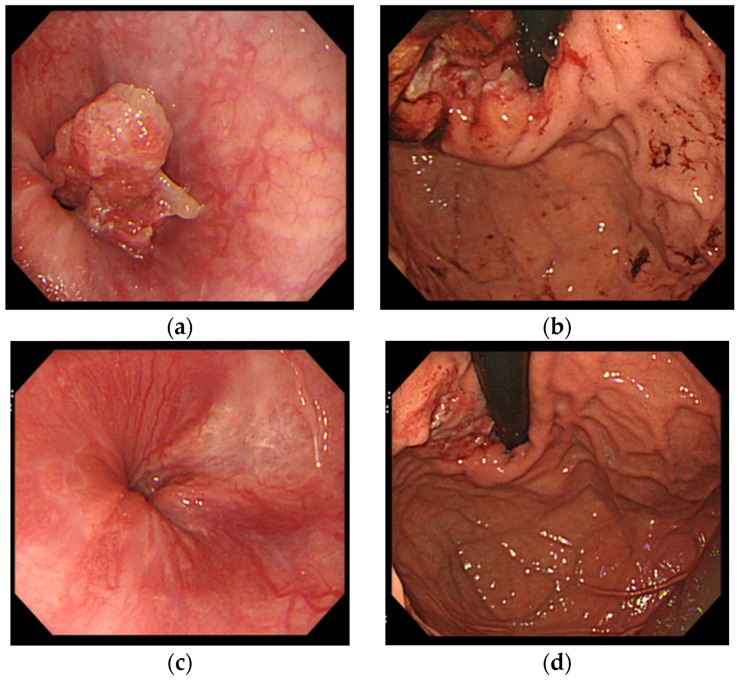
Gastrointestinal fiberscope (GIF) revealed esophageal invasion (**a**) and an advanced gastric cancer type 3 in cardia (**b**) before the initiation of chemo-immunotherapy. After four cycles of chemo-immunotherapy in combination with trastuzumab, esophageal tumor completely disappeared, leaving a whitish area (**c**), and both round wall and ulceration became flattened (**d**).

In a 42-year old man presented with remarkable hepatomegaly, liver dysfunction accompanied with a mild degree of jaundice was observed; AST 107 U/L (13–33), ALT 79 U/L (6–30), ALP 1198 U/L (115–359), LDH 1413 U/L (119–229), total bilirubin 2.1 mg/dL, CEA 4.3 ng/mL (<5.0), and CA19-9 14.6 U/mL (<37.0). Since a CT scan revealed multiple liver tumors and lymph node swelling (Figure 1a,b), he was consulted by our institution. Advanced gastric cancer type 3 with esophageal invasion was diagnosed by gastrointestinal fiberscope (Figure 2a,b). Since immune-histochemical examination of biopsy samples revealed overexpression of HER2, scored as 3+, trastuzumab was administered for the treatment of gastric cancer. After four cycles of chemo-immunotherapy comprising S-1, plus cisplatin, lentinan, and trastuzumab, re-evaluation was made, showing a good reduction of the metastatic liver tumors (Figure 1c,d) as well as the primary gastric lesion (Figure 2c,d). After two cycles of PSC triple therapy combined with lentinan and trastuzumab, further decrease in both primary and liver lesions was demonstrated. Consequently, the chemotherapeutic efficacy was diagnosed as PR.

## 5. Discussion

Five of 12 gastric cancer patients with extensive liver metastases showed an objective response to chemo-immunotherapy using lentinan. The median OS of 12 cases exceeded one year, which is fairly good, considering that these cases had multiple liver metastases. The responders survived significantly longer than non-responders. As for the HER2 status, three patients showed an objective response among 10 individuals with its low expression and only two cases (CR, 1; PR, 1) survived, while eight other cases had already died within the two years following the initiation of original chemotherapy. In contrast, two patients with high HER2 expression, who were still on trastuzumab in combination with chemo-immunotherapy, revealed good PR. Molecular targeting agents are known to be useful for the treatment of gastric cancer [19,20], and the chemo-sensitivity difference in our cases should be associated with their status of HER2 expression. It has been reported that trastuzumab, a humanized IgG1 antibody specific for the cellular proto-oncogene HER2/neu, mediates antibody dependent cellular cytotoxicy (ADCC) [21]. The binding of lentinan to leukocytes could induce IL-12 production [10,22] and enhance the anti-tumor effects of monoclonal antibodies through augmented ADCC [23]. Considering these properties of lentinan, its synergistic action with targeting cancer therapy might be responsible for the therapeutic effects.

Recently, there has been an increasing amount of evidence of sustained tumor regression in patients with melanoma and non-small-cell lung cancer after treatment with immunotherapies targeting immune checkpoints such as programmed cell death-1 ligand 1 (PD-L1) [24,25]. PD-L1 expression has been observed in a variety of solid tumors including gastric cancer [26,27], which engages programmed cell death-1 (PD-1) on T cells and subsequently triggers inhibitory signaling downstream of the T-cell antigen receptors, reducing T-cell killing capacity [28]. Some chemotherapeutic agents can affect PD-L1 expression in tumor cells [29,30]. Increased PD-L1 expression on cancer cells can be an important escape mechanism from the host T cell immunity [31]. We suspect that lentinan inhibit the overexpression of PD-L1 caused by cisplatin based on our preliminary experiments. An *in vitro* study may help provide clarity on the contribution of lentinan to the elimination of gastric cancer cells through potentiating host immune response. Lentinan was also reported to decrease prostaglandin (PG) E2 secretion [32]. Immunosuppressive properties of PGE2 are associated with inactivation of T cells and antigen-presenting cells, causing cancer progression. Lentinan can enhance the chemotherapeutic effects in drug refractory tumor microenvironment, which might lead to tumor clearance.

## 6. Conclusions

Lentinan serves synergistic actions with a molecular targeting agent and cytotoxic drugs through the modulation of ADCC or PD-1/PD-L1 axis, which may support the idea that the chemo-immunotherapy prolongs the survival of metastatic gastric cancer patients, compared to chemotherapy alone.

## Figures and Tables

**Table 1:** Characteristics of gastric cancer patients with liver metastases receiving lentinan.

Characteristics		Number of Patients
Gender	Male	8
	Female	4
Age (years)	Range	42–82
	Median	67
Performance status	0	5
	1	3
	2	4
HER2 status	High	2
	Low	10
Target lesions	Primary	9
	Liver	12
	Lung	2
	Peritoneum	4
	Lymph nodes	12
Original chemotherapy	S-1 alone	1
	S-1/cisplatin	8
	PSC	3

PSC: paclitaxel, S-1, and cisplatin.

**Table 2:** Patients’ list.

Case	Age	Gender	HER2	S-1	Cisplatin	Taxanes	Trastsuzumab	Start	Last	OR	Outcome
1	65	Male	Low	+	+	+	−	07.09.2010	31.01.2016	CR	Alive
2	67	Female	Low	+	+	−	−	21.05.2015	31.01.2016	PR	Alive
3	74	Female	Low	+	+	+	−	07.12.2011	06.11.2013	PR	Dead (liver failure)
4	71	Male	High	+	+	+	+	18.09.2015	31.01.2016	PR	Alive
5	42	Male	High	+	+	+	+	07.07.2015	31.01.2016	PR	Alive
6	58	Male	Low	+	+	+	−	01.10.2010	29.07.2012	SD	Dead (obstructive jaundice)
7	67	Male	Low	+	+	−	−	27.12.2013	21.11.2014	SD	Dead (liver failure)
8	52	Female	Low	+	+	+	−	27.02.2013	22.09.2013	SD	Dead (meningitis)
9	76	Male	Low	+	+	−	−	11.09.2014	31.12.2015	SD	Dead (liver failure)
10	75	Male	Low	+	+	+	−	08.03.2014	19.04.2015	SD	Dead (liver failure)
11	82	Male	Low	+	−	−	−	02.03.2011	13.03.2012	PD	Dead (liver failure)
12	72	Female	Low	+	+	−	−	07.12.2011	23.02.2012	PD	Dead (multiple organ failure)

HER2: human epidermal growth factor receptor 2; Start: the date of the initiation of chemotherapy; Last: the date of death or the most recent follow-up day; OR: objective response; CR: complete response; PR: partial response; SD: stable disease; PD: progressive disease.

**Table 3:** Patients with liver metastases showing complete response in the literature.

Age	Gender	Before	Original Regimen	CR Duration (Months)	Recurrence
72	Male	T4 N3 H3	Paclitaxel/Doxifluridine	14	+
56	Male	T4 N3 H1	S-1/Cisplatin	84	−
48	Male	T4 N1 H1	FOLFOX4	43	−
65	Female	T4 NX H1	DOX	9	+
65	Male	T4 N3 H3	PSC plus lentinan	33	−

H1: metastasis limited to 1 hepatic lobe; H2: scattered metastases in both lobes; H3: multiple metastases in both lobes; DOX: docetaxel, oxaliplatin, and capecitabine; PSC: paclitaxel, S-1, and cisplatin.
